# Interpretable machine learning approach to analyze the effects of landscape and meteorological factors on mosquito occurrences in Seoul, South Korea

**DOI:** 10.1007/s11356-022-22099-5

**Published:** 2022-07-28

**Authors:** Dae-Seong Lee, Da-Yeong Lee, Young-Seuk Park

**Affiliations:** Department of Biology, Kyung Hee University, Seoul, 02447 Republic of Korea

**Keywords:** Mosquito occurrence pattern, Prediction model, Landscape conditions, Meteorological conditions, Shapley additive explanations

## Abstract

Mosquitoes are the underlying cause of various public health and economic problems. In this study, patterns of mosquito occurrence were analyzed based on landscape and meteorological factors in the metropolitan city of Seoul. We evaluated the influence of environmental factors on mosquito occurrence through the interpretation of prediction models with a machine learning algorithm. Through hierarchical cluster analysis, the study areas were classified into waterside and non-waterside areas, according to the landscape patterns. The mosquito occurrence was higher in the waterside area, and mosquito abundance was negatively affected by rainfall at the waterside. The mosquito occurrence was predicted in each cluster area based on the landscape and cumulative meteorological variables using a random forest algorithm. Both models exhibited good performance (both accuracy and AUROC > 0.8) in predicting the level of mosquito occurrence. The embedded relationship between the mosquito occurrence and the environmental factors in the models was explained using the Shapley additive explanation method. According to the variable importance and the partial dependence plots for each model, the waterside area was more influenced by the meteorological and land cover variables than the non-waterside area. Therefore, mosquito control strategies should consider the effects of landscape and meteorological conditions, including the temperature, rainfall, and the landscape heterogeneity. The present findings can contribute to the development of mosquito forecasting systems in metropolitan cities for the promotion of public health.

**Supplementary Information:**

The online version contains supplementary material available at 10.1007/s11356-022-22099-5.

## Introduction

Mosquitoes are notorious insect species that pose a major threat to human health, killing 725,000 people annually (World Health Organization 2018). Mosquito-associated mortality is attributed to their role as vectors for disease transmission. Among the various mosquito species, three genera are known to be the main vectors of viral diseases (World Health Organization 2018). *Aedes* causes Chikungunya, dengue fever, and carries the Zika virus. *Anopheles* causes malaria, and *Culex* is known as a vector for Japanese encephalitis and West Nile fever. These mosquito-borne diseases have spread worldwide, posing a global threat to human health (Gubler et al. 2001; Kindhauser et al. 2016).

Various studies have been conducted on mosquitoes (Hales et al. 2002; Hay et al. 2002; Kearney et al. 2009; Lebl et al. 2013; Maguire et al. 1999; Sutherst 2004). The distribution and occurrence of mosquitoes are determined by various factors, including human activities such as travel and trade (Reiter 2001) as well as changes in the habitat, temperature, and rainfall (Epstein 2002; Ruiz et al. 2010, Vandyk and Rowley 1995). An increase in temperature and rainfall reduction can induce the occurrence of mosquito-borne diseases (Ruiz et al. 2010). Benedum et al. (2018) previously developed a statistical model for the effect of rainfall flushing on dengue transmission in Singapore.

With recent advances in computational science, machine learning methods are frequently employed in the study of mosquito occurrence. Machine learning algorithms have been used to understand and predict various ecological phenomena (Olden et al. 2008). Such algorithms have been used for the selection of important variables for mosquito distribution among climate data (Wieland et al. 2017). Decision trees and random forest models have also been employed to predict mosquito distribution (Früh et al. 2018; Kwon et al. 2015). Artificial neural networks for the prediction of mosquito abundance and mosquito-borne disease incidence have also been previously described (Laureano-Rosario et al. 2018; Lee et al. 2016). Studies on mosquito control have also implemented maximum entropy modeling and artificial neural networks (Joshi and Miller 2021). Based on these studies, mosquito forecasting systems have been implemented as part of public health programs in various countries. For example, the Northeast Regional Climate Center in the USA forecasted a potential mosquito population in the Northeastern United States (Gong et al. (2011); http://www.nrcc.cornell.edu/industry/mosquito/). In Southeast Asia, a dengue forecasting model satellite-based system (D-MOSS) was constructed to develop a dengue fever early warning system (Colón-González et al. 2021).

Various interpretable machine learning methods have recently been developed to overcome weaknesses in machine learning algorithms which are a lack of interpretability and causality occurring between the input and the output values of the models (Arrieta et al. 2020, Das and Rad 2020). Among them, the partial dependence plot (PDP) (Friedman 2001; Greenwell et al. 2018) has been widely used to graphically visualize non-linear, non-monotonous responses of model outputs to changes in an independent variable (Lee et al. 2021a, 2019). Shapley additive explanation (SHAP) (Lundberg and Lee 2017) is frequently used to evaluate the contribution of input variables to an individual prediction because of its distinctive advantages such as clear interpretation, easy implementation, and it being applicable to any type of data (Cha et al. 2021; Linardatos et al. 2021; Molnar 2022; Razavi et al. 2021).

South Korea is among the countries under constant threat of mosquito-borne disease. Korean patients often present with mosquito-transmitted malaria and Japanese encephalitis. In particular, the number of patients infected with Japanese encephalitis has steadily increased since 2017 (Korea Centers for Disease Control and Prevention 2019). Therefore, many studies have been conducted in South Korea on mosquito distribution (Jeong and Lee 2003, Lee and Hong 1995) and the relationship between mosquitoes and environmental conditions, including meteorological and spatial factors (Chae et al. 2014, Kim and Park 2013, Shin 2011). A locally developed mosquito forecasting system was recently implemented in Seoul, the metropolitan capital of Korea (Park and Im 2018), and is to be implemented throughout the country.

However, urban areas such as Seoul Metropolitan City have different mosquito occurrence characteristics from the natural environment, because many factors such as landscape, land use, meteorological factors, and mosquito control activities affect the occurrence of mosquitos. Therefore, to effectively evaluate the mosquito occurrence in urban areas, this information must be considered. However, there are limited studies that reveal the relationship between mosquitoes and the urban environment.

The present study was conducted to support the Korean mosquito forecasting system and to suggest relevant predictive variables in urban areas through interpretable machine learning approaches. We analyzed the patterns of mosquito occurrence in relation to meteorological factors and employed a machine learning algorithm to predict the occurrence of urban mosquitoes with interpretable methods.

## Materials and methods

### Mosquito abundance

Mosquito abundance data were obtained from the Public Health Center of Yeongdeungpo-gu, Seoul, South Korea. Seoul (centroid: longitude 126.992, latitude 37.552) is the capital of South Korea with a basin topography and is located in the midwest of the Korean Peninsula. According to the Köppen–Geiger climate classification system, the Seoul climate is a humid continental climate with snow, dry winters, and hot summers (Kottek et al. 2006) (In Seoul, the annual average temperature is 12.5 ℃, and annual precipitation is 1417.9 mm). The Han River also flows through Seoul. Yeongdeungpo-gu (centroid: longitude 126.910, latitude 37.522) is an administrative district located south of the Han River in Seoul. It is mostly flat land, with many factories and residences.

**Fig. 1:**
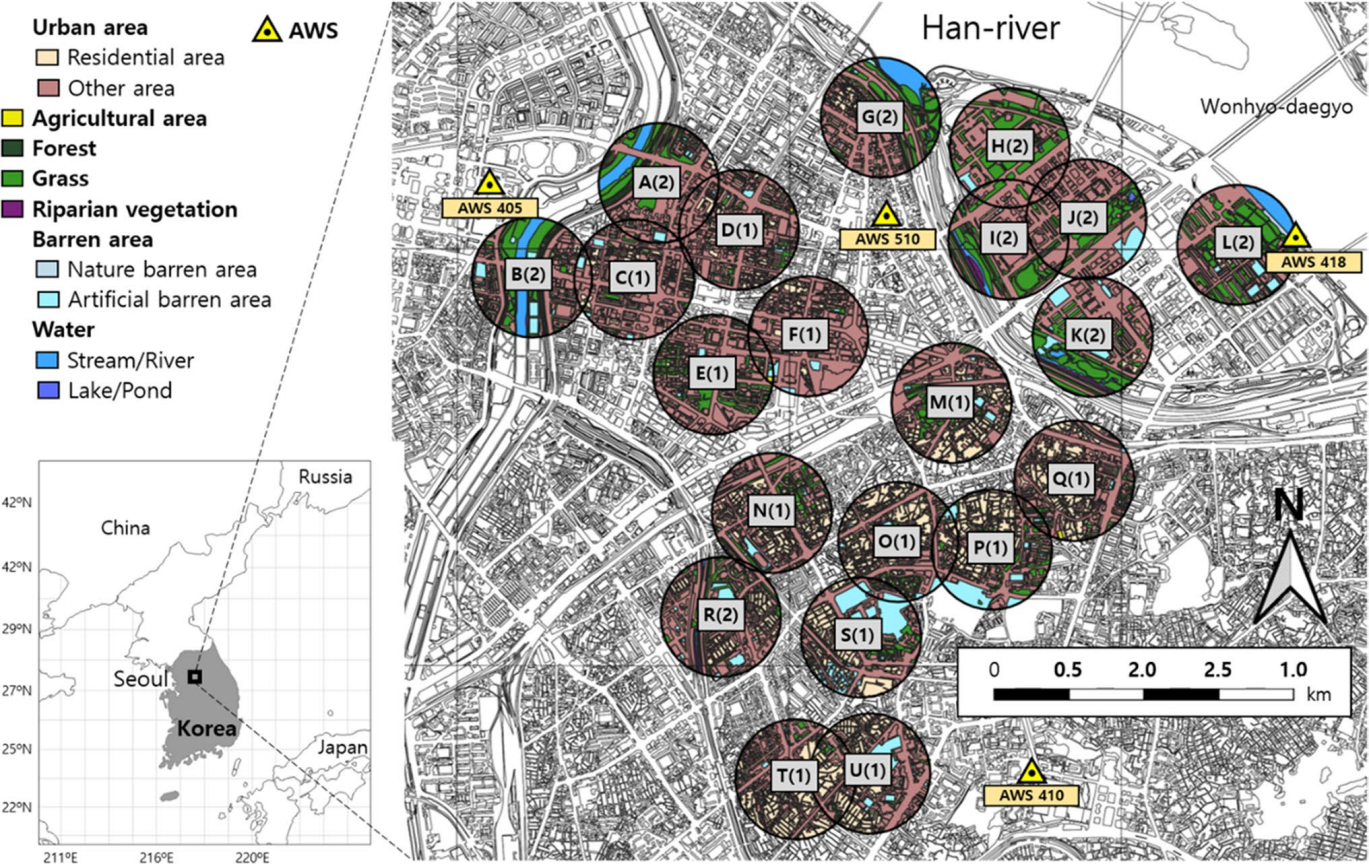
Location of 21 mosquito monitoring sites (A − U) in Yeongdeungpo-gu, Seoul, South Korea. Colors within circles show each land cover type within a radius of 400 m. The number in parenthesis indicates a cluster number defined in the hierarchical cluster analysis (Fig. 2). Four automatic weather stations were located in the study area

The mosquito abundance was measured daily using automatic collecting machines, which form part of the digital mosquito monitoring system (DMS, Environmental Technology & Development; http://www.etnd.co.kr/). These were placed at 21 monitoring sites (Fig. 1) during the night (6:00 pm–7:00 am) from May to October over 5 years (2011–2015) (Kwon et al. 2015). The mosquito data for four of these years (2011–2013, 2015) were used in this study. The data from 2014 were excluded because of many unexpected values caused by problems with the DMS calibration.

The DMS attracted female mosquitoes by diffusing carbon dioxide (300 mL/min) and automatically counted the mosquitoes passing through the observation cage using an infrared beam sensor. The data were sent to the computer server via a code division multiple access method in real time and then stored on the database server. The DMS was effective in counting the mosquito abundance, with a high correlation coefficient (*r* > 0.92) between the manual and automatic counts of the mosquito numbers (Kwon et al. 2015; Yi et al. 2014). Abnormal mosquito abundance values, namely, unrealistic values, were attributed to mechanical malfunction or other organisms, such as spiders, and were excluded from the analysis. Days with more than 300 individuals being counted were considered outliers based on the distribution of the total daily mosquito abundance, consultation with experts, and the literature (Kwon et al. 2015). Outliers or missing values were replaced with an average value between the previous and the following days.

The DMS could not distinguish species in the data transmitted because it only counted the number of mosquitoes passing through the observation cage. According to mosquito monitoring data collected with light traps from 2011 to 2017 in Seoul, Korea (https://news.seoul.go.kr/welfare/mosquito), 13 species were recorded, with *Culex pipiens pallens* accounting for 92.5% of the total abundance, followed by *Aedes koreicus* (5.3%) and *Aedes vexans nipponii* (1.0%). Although there was annual variation in the total abundance of other species, their abundance was less than 1.0% of the total. Therefore, mosquito abundance in this study mainly represents the abundance of *C. pipiens pallens*, which is the dominant mosquito species in Seoul.

### Environmental data

Meteorological data including the daily average temperature, daily minimum temperature, daily maximum temperature, daily rainfall, and the number of rainfall days were obtained using automatic weather stations (AWSs) operated by the Korea Meteorological Administration (https://data.kma.go.kr/). The four AWSs (AWS 405, 410, 418, and 510) were located in the study area (Fig. 1), and the meteorological data measured at the AWS nearest to the mosquito monitoring site were used in the study (AWS 405, sites A, B, and C; AWS 410, sites O, P, Q, R, S, T, and U; AWS 418, sites K and L; AWS 510, sites D, E, F, G, H, I, J, M, and N). During the study period from May to October (Table 1), the daily average temperature (± standard deviation) was 22.5 °C (± 4.6), the minimum temperature was 18.8 °C (± 5.0), the maximum temperature was 27.0 °C (± 4.7), and the rainfall was 6.1 mm (± 21.2), respectively. Meteorological data for 60 cumulative days (1, 2, ~ , 60 days) were used to create a cumulative meteorological dataset. For example, a cumulative five days of rainfall on June 10 represented the cumulative rainfall for the five days from June 5th to June 9th.

**Table 1:** Summary of meteorological data (mean ± standard deviation) measured at each automatic weather station (AWS) during the study period

Variables		Weather station				Mean
	Year	AWS 510	AWS 418	AWS 410	AWS 405	
Daily average temperature (°C)	2011	21.8 ± 4.6	21.9 ± 4.5	21.3 ± 4.5	21.8 ± 4.7	21.7 ± 4.6
	2012	22.8 ± 4.6	23.1 ± 4.6	22.3 ± 4.6	22.8 ± 4.7	22.7 ± 4.6
	2013	22.8 ± 4.8	22.8 ± 4.7	22.3 ± 4.7	22.8 ± 4.9	22.7 ± 4.8
	2015	22.9 ± 4.3	22.8 ± 4.3	22.4 ± 4.3	22.8 ± 4.4	22.7 ± 4.3
	Mean	22.6 ± 4.6	22.7 ± 4.6	22.1 ± 4.5	22.6 ± 4.7	22.5 ± 4.6
Daily minimum temperature (°C)	2011	18.4 ± 5.0	18.3 ± 4.9	17.7 ± 5.1	18.1 ± 5.2	18.1 ± 5.0
	2012	19.4 ± 4.8	19.5 ± 4.8	18.5 ± 4.8	19.2 ± 4.9	19.1 ± 4.8
	2013	19.4 ± 5.3	19.3 ± 5.1	18.6 ± 5.3	19.2 ± 5.4	19.1 ± 5.3
	2015	19.2 ± 4.5	19.2 ± 4.5	18.1 ± 4.6	18.8 ± 4.7	18.9 ± 4.6
	Mean	19.1 ± 4.9	19.1 ± 4.8	18.2 ± 5.0	18.8 ± 5.1	18.8 ± 5.0
Daily maximum temperature (°C)	2011	26.0 ± 4.7	26.4 ± 4.8	25.6 ± 4.6	26.1 ± 4.8	26.0 ± 4.7
	2012	27.1 ± 4.8	27.7 ± 4.8	26.7 ± 4.7	27.3 ± 4.8	27.2 ± 4.8
	2013	27.1 ± 4.7	27.0 ± 4.8	26.8 ± 4.5	27.4 ± 4.8	27.1 ± 4.7
	2015	27.5 ± 4.5	27.3 ± 4.6	27.4 ± 4.5	27.9 ± 4.6	27.5 ± 4.5
	**Mean**	26.9 ± 4.7	27.1 ± 4.8	26.6 ± 4.6	27.2 ± 4.8	27.0 ± 4.7
Daily rainfall (mm)	2011	9.0 ± 31.5	8.5 ± 28.2	9.5 ± 30.2	7.9 ± 29.5	8.7 ± 29.8
	2012	7.2 ± 22.9	6.7 ± 22.1	7.7 ± 25.2	7.2 ± 22.1	7.2 ± 23.1
	2013	5.6 ± 17.3	5.7 ± 16.9	5.5 ± 18.0	5.1 ± 15.7	5.5 ± 17.0
	2015	3.1 ± 8.9	3.0 ± 9.0	2.6 ± 7.9	3.0 ± 8.7	2.9 ± 8.6
	Mean	6.2 ± 21.8	6.0 ± 20.4	6.3 ± 22.1	5.8 ± 20.5	6.1 ± 21.2

The landscape data for the monitoring sites were obtained from a digital map provided by the Environmental Geographic Information Service in Korea (https://egis.me.go.kr) through extracting land coverage (%) within a 400-m radius using a geographic information system (QGIS 3.12; QGIS.org (2021)) (Fig. 1). This digital land use map was constructed using an aerial orthophotograph of Korea (0.25 × 0.25 m^2^ resolution), and the land cover was categorized into a total of 41 classes by the Ministry of Environment, Korea. In this study, we used 13 land cover classes (seven major classes, urban area, agriculture land, forest, grass area, barren area, wetland, and water; and six detailed classes:,esidential and other areas in the urban area, nature and artificial area in a barren area, stream/river, and lake/pond in water). Distance to the rivers was also calculated for each DMS monitoring site. The radius was chosen according to the average flight distance (402 m) of *C. pipiens pallens* (Verdonschot and Besse-Lototskaya 2014), which is the dominant species in this area.

### Data analysis

**Fig. 2:**
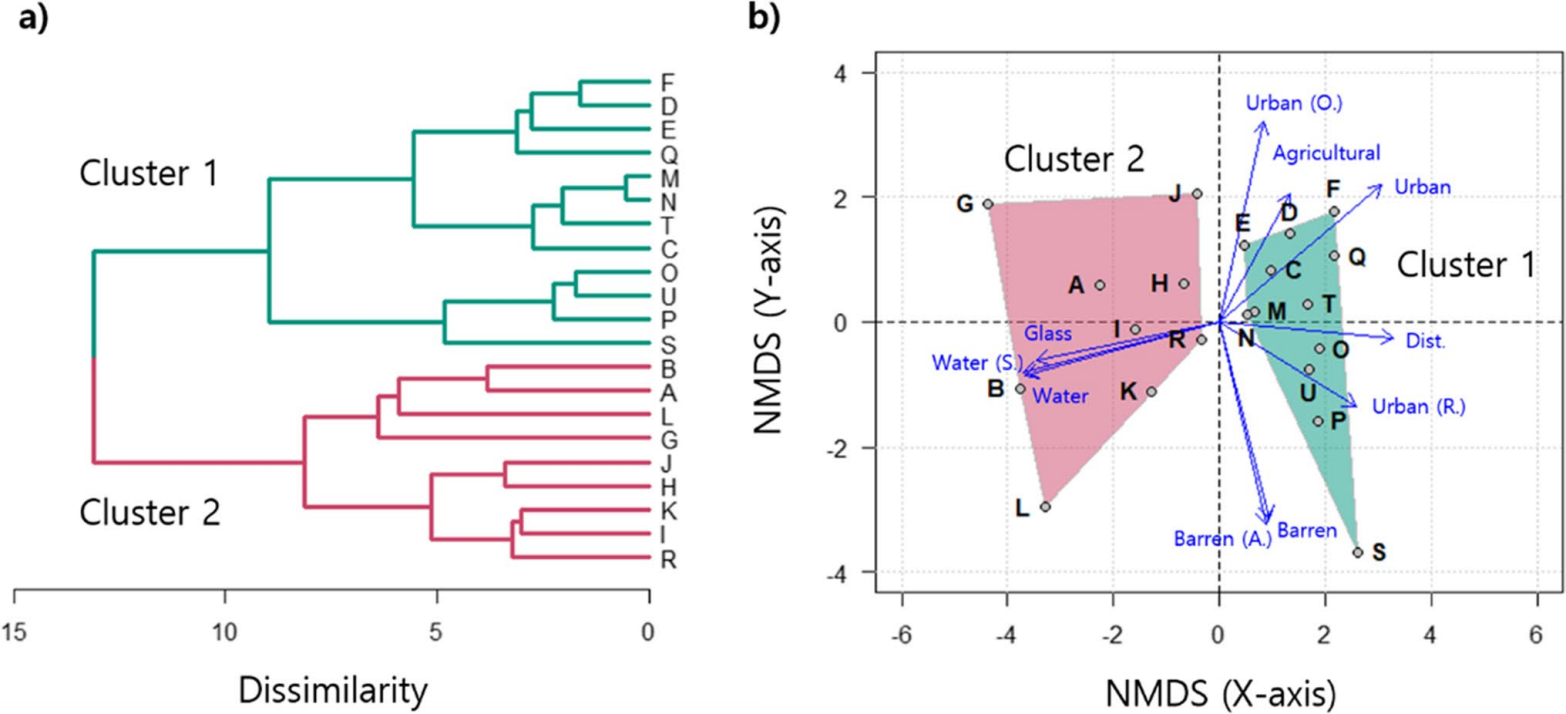
**a** Classification of mosquito monitoring sites based on environmental conditions with a hierarchical cluster analysis via Euclidean distance and Ward linkage method. **b** Ordination of monitoring sites on non-metric multidimensional scaling with Euclidean distance. Only some variables showing statistically significant differences were displayed. Abbreviations for landscape variables are given in Table 3

The habitat conditions of mosquitoes are important for determining their occurrence (Asigau and Parker 2018, Montagner et al. 2017). Therefore, 21 mosquito monitoring sites in the study were classified based on their landscape conditions with land coverage being divided into 13 classes using hierarchical cluster analysis (HCA) with a Euclidean distance measure and the Ward linkage method (Fig. 2). The land cover data were standardized prior to the HCA. The same data were used for non-metric multidimensional scaling (NMDS), which was conducted to analyze the relationships among the monitoring sites according to the landscape conditions.

The effects of the meteorological factors on mosquito occurrence were evaluated using Spearman rank correlation analysis and the Mann–Whitney *U* test. Cumulative meteorological data were used to explain the time delay effect of meteorological factors on the mosquito abundance instead of time-lagged data because the cumulative meteorological data showed a stronger correlation with the mosquito abundance than the time-lagged meteorological variables.

We analyzed the effects of the meteorological factors on mosquito occurrence and abundance. The mosquito abundance, difference in abundance, and the change rate (%) of abundance were compared during meteorological events such as rainy weather (continuous rainy days) and dry weather (more than two days without rain) using the pairwise Mann–Whitney *U* test.

### Prediction model for mosquito occurrence and evaluation

A model using a random forest algorithm (RF), which is a machine learning method, was developed to predict the daily mosquito occurrence under two different landscape conditions defined in the HCA. RF is a useful method for the classification and prediction of ecological data with a high level of accuracy (Cutler et al. 2007; Lee et al. 2020). In the previous study (Kwon et al. 2015) conducted at the same study sites, the RF model showed a better performance in predicting the mosquito occurrence than other machine learning methods such as the support vector machine and the classification and regression tree. Therefore, we used the RF model as a prediction model algorithm in this study. During the modeling process, the mosquito abundance at each monitoring site was categorized into four levels according to the criteria of the mosquito forecast system in Seoul, South Korea (http://news.seoul.go.kr/welfare/mosquito) (Table 2).

**Table 2:** Definition of mosquito levels and intervals in the mosquito forecast system in Seoul, Korea

Level	Range (n*)	Description
1	0 ≤ *n* < 30	Comfortability
2	30 ≤ *n* < 60	Attention
3	60 ≤ *n* < 90	Caution
4	90 ≤ *n* ~	Displeasure

^*^Number of mosquitoes (Individual/day per station)

To exclude multicollinearity among the meteorological and landscape variables, only variables that were selected based on a variance inflation factor (VIF; VIF < 5) were used in the RF models for the different clusters defined in the HCA. Therefore, the dependent variables were different between clusters. In the RF models, the daily mosquito level was set as the dependent variable, whereas the selected meteorological and landscape variables were used as independent variables (Table 3). Among the 25 independent variables selected, nine variables including the month (mosquito occurrence date) and the cumulative rainfall for 1 and 4 days were used in both models of the two clusters defined in the HCA; eight variables including the cumulative minimum temperature for 1 and 56 days, as well as the cumulative rainfall for 12 and 60 days, were only used in the RF model for cluster 1. Eight variables, including the cumulative minimum temperature for 13 days and the cumulative rainfall for 2 and 14 days, were used in the RF model for cluster 2.

**Table 3:** Environmental variables considered in the study. Variables used in the mosquito occurrence prediction via random forest models. Numbers (1–60) following variable names indicate the cumulative days for each corresponding variable. Only variables selected based on the variance inflation factor (VIF; VIF > 5) were used in the prediction models

Category		Variable	Abbreviation	Model for Cluster 1	Model for Cluster 2
Dependent	Mosquito	Mosquito levels (1, 2, 3, 4)		√	√
Independent	Time	Month	Month	√	√
	Meteorology	Cumulative minimum temperature 1	Tmin 1	√	
		Cumulative minimum temperature 13	Tmin 13		√
		Cumulative minimum temperature 56	Tmin 56	√	
		Cumulative rainfall 1	Rain 1	√	√
		Cumulative rainfall 2	Rain 2		√
		Cumulative rainfall 4	Rain 4	√	√
		Cumulative rainfall 12	Rain 12	√	
		Cumulative rainfall 14	Rain 14		√
		Cumulative rainfall 60	Rain 60	√	
		Cumulative number of rainy day 1	Rday 1	√	√
		Cumulative number of rainy days 2	Rday 2	√	√
		Cumulative number of rainy days 5	Rday 5	√	√
		Cumulative number of rainy days 31	Rday 31		√
		Cumulative number of rainy days 43	Rday 43	√	
		Cumulative number of rainy days 60	Rday 60		√
	Land coverage	Agricultural area	Agriculture	√	
		Barren area (total)	Barren	√	√
		Barren area (nature)	Barren (N.)	√	√
		Barren area (artificial)	Barren (A.)		
		Forest area	Forest		√
		Grass area	Glass	√	
		Riparian vegetation	Riparian		√
		Urban area (total)	Urban		√
		Urban area (residential)	Urban (R.)	√	√
		Urban area (other)	Urban (O.)		
		Water	Water		
		Water (stream)	Water (S.)		
		Water (pond)	Water (P.)		
	Geography	Distance to rivers	Dist	√	

Two RF models were developed for each cluster, and the mosquito occurrence at each monitoring site was predicted for each cluster. For each cluster, the datasets were divided into two subsets for training and testing the models at a ratio of 7:3 by considering the monitoring sites and the mosquito occurrence levels. The accuracy (ACC) and the area under the receiver operating characteristic (AUROC) curve value were used to evaluate the performance of the RF models for the mosquito occurrence levels. In this study, ACC and AUROC curve value mean balanced accuracy and multi-class AUROC curve value are suitable indices for multi-class datasets (Grandini et al. 2020, Hand and Till 2001, Sokolova and Lapalme 2009). There is no universal evaluation value for ACC. However, empirically, when ACC is 0.8 or more, it is considered to be high accuracy. If the AUROC curve value of the model is more than 0.7, the model is considered to have a good performance for prediction (Greiner et al. 2000; Ray et al. 2010).

### Evaluation of variables on the mosquito occurrence

In this study, we used SHAP to interpret the RF models developed. The SHAP approach is an emerging methodology to identify and explain the internal structures of the black-box model such as machine learning models, providing strong interpretability (Abdollahi and Pradhan 2021, Cha et al. 2021, Lundberg and Lee 2017). The SHAP value of the SHAP method is the conditional Shapley value based on game theory and represents the quantitative contribution of each variable to response variable prediction (Lundberg and Lee 2017).

Using the SHAP method, the importance of variables in mosquito occurrence and the relationship between the mosquito occurrence levels and the dependent variables (meteorological and landscape variables) in the RF models were evaluated through variable importance and partial dependence plots (PDPs). In this evaluation, SHAP values are used as a variable contribution instead of Gini importance in the importance calculation, and as the average response to the independent variables in PDPs (Liaw and Wiener 2002, Molnar 2022). Therefore, a high and positive SHAP value indicates that the variable highly and positively affects the output of the prediction model. In this study, the SHAP value was calculated using the kernel SHAP method.

All the analyses and the statistical tests used in this study were conducted using the R program (R Core Team 2021) with specific packages. The packages “stats” (R Core Team 2021) and “dunn.test” (Dinno 2017) were used for non-parametric statistics tests (the Mann–Whitney *U* test, Kruskal–Wallis test, and Dunn’s test) and HCA. The package “vegan” (Oksanen et al. 2020) was used for NMDS, the package “randomForest” (Liaw and Wiener 2002) was used for the RF modeling, the package “pROC” (Robin et al. 2011) was used to calculate the multiclass AUROC value, and the package “shapper” (Maksymiuk et al. 2020) was used to calculate the SHAP value.

## Results

### Characteristics of mosquito occurrence and relationship with the environment

The 21 monitoring sites were classified into two clusters via HCA, based on the environmental conditions (Fig. 2). According to the NMDS, cluster 1 was mainly affected by the proportion of urban (total urban, residential, and other) areas, agricultural areas, and the distance to the rivers from the monitoring site, whereas cluster 2 was strongly influenced by the proportion of water, stream, and grass areas.

**Fig. 3:**
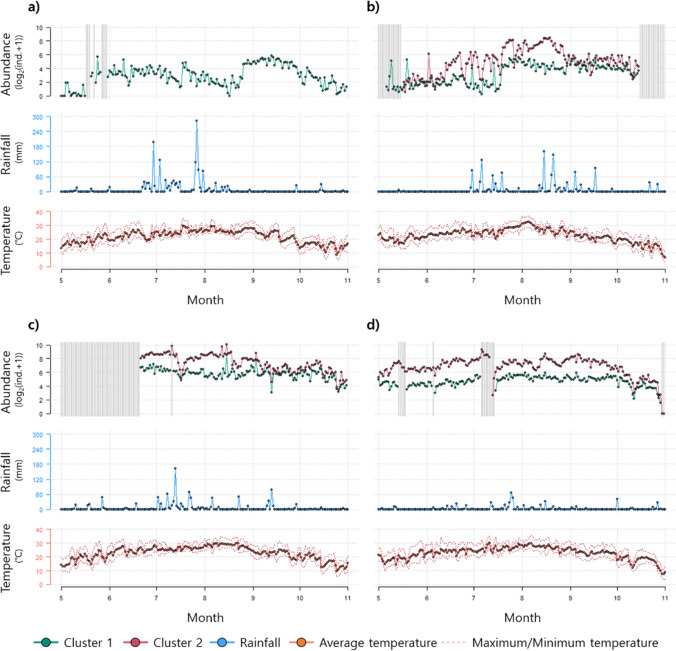
Changes in the daily mosquito abundance and the meteorological factors (temperature and rainfall) in 2011 (**a**), 2012 (**b**), 2013 (**c**), and 2015 (**d**). The mosquito abundance was averaged with values of each site for each cluster. The temperature and rainfall are presented as the average values for four automatic weather stations. Gray area and vertical lines indicate the time when the digital mosquito monitoring system was not operated

Changes in the daily mosquito abundance in each cluster and the meteorological factors (temperature and rainfall) were analyzed (Fig. 3). Mosquito data from 2011 were not available for cluster 2 because the DMS for this cluster operated since 2012 (Fig. 3a). The daily mosquito abundance was generally high in the summer and autumn for both clusters. Rainfall was also high in the summer and autumn, with heavy rain occurring mainly from July to September. The annual rainfall amount decreased from 1645.5 mm in 2011 to 531.8 mm in 2015. The daily mosquito abundance was significantly different between the two clusters (Mann–Whitney *U* test, *p* < 0.001), with a higher abundance in cluster 2 than in cluster 1.

**Fig. 4:**
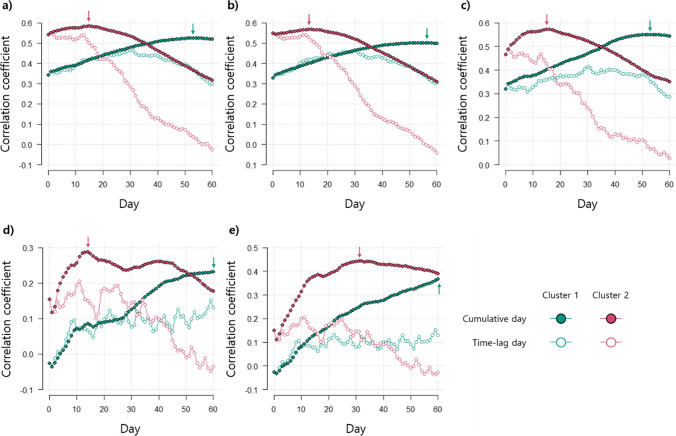
Changes in the Spearman rank correlation between the mosquito abundance and the environmental factors according to the difference in cumulative or time-lag days for each cluster. **a** Average temperature, **b** minimum temperature, **c** maximum temperature, **d** rainfall, and **e** number of rainy days. The x-axis presents the cumulative number of days and lagged number of days for each meteorological variable. The arrows in the figure present the day with maximum correlation coefficients in each cluster

The average mosquito abundance for both clusters displayed a positive correlation with most cumulative meteorological factors, particularly with cumulative meteorological factors (*r* = 0.092–0.585, *p* < 0.05), to a greater extent than with the time-lagged meteorological factors (*r* = 0.079–0.551, *p* < 0.05) (Fig. 4). Therefore, cumulative meteorological variables were used in the RF modeling to predict the mosquito occurrence. The mosquito abundance for cluster 1 had the strongest correlation with the cumulative average temperature for 53 days (*r* = 0.525, *p* < 0.001), the cumulative minimum temperature for 56 days (*r* = 0.502, *p* < 0.001), the cumulative maximum temperature for 53 days (*r* = 0.551, *p* < 0.001), the cumulative rainfall for 60 days (*r* = 0.233, *p* < 0.001), and the cumulative number of rainy days for 60 days (*r* = 0.367, *p* < 0.001). However, the mosquito abundance in cluster 2 had the highest correlation coefficient with the cumulative average temperature for 15 days (*r* = 0.585, *p* < 0.001), the cumulative minimum temperature for 13 days (*r* = 0.568, *p* < 0.001), the cumulative maximum temperature for 15 days (*r* = 0.574, *p* < 0.001), the cumulative rainfall for 14 days (*r* = 0.288, *p* < 0.001), and the cumulative number of rainy days for 31 days (*r* = 0.445, *p* < 0.001).

**Fig. 5:**
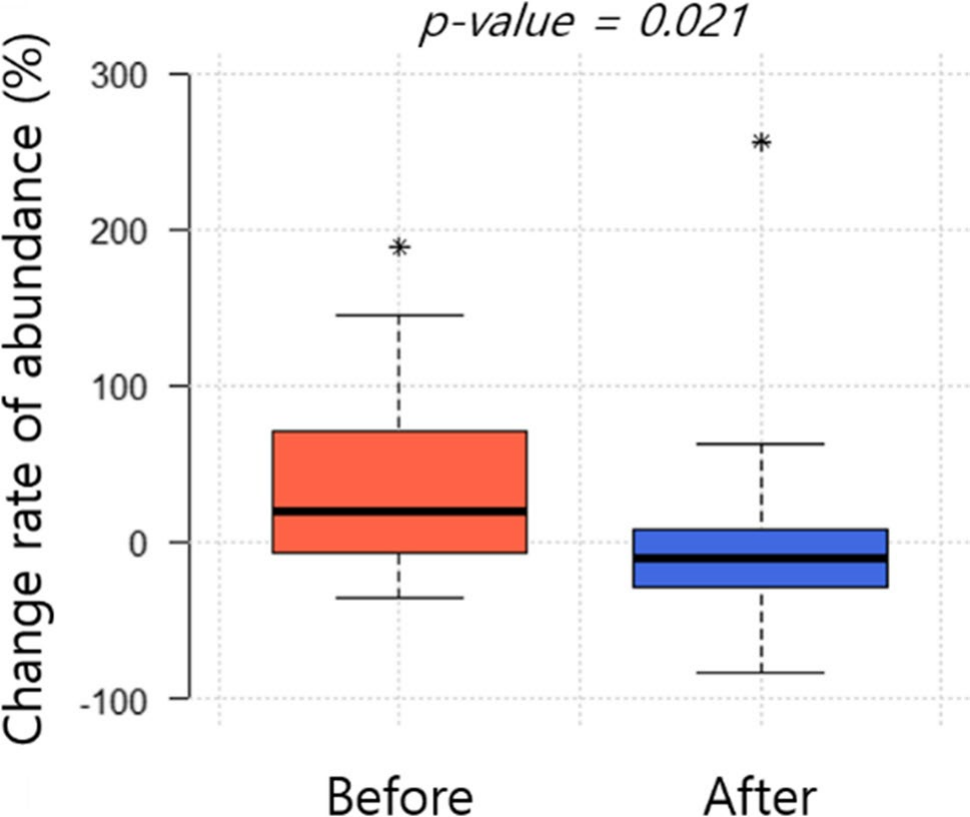
Change rate (%) of the mosquito abundance before and after a rainy weather in cluster 2. The pairwise Mann–Whitney *U* test was used for the statistical analysis. When the rain did not last for more than 4 days before rainy weather, a statistically significant difference was observed in the mosquito change rate. Asterisks mean outliers

During the study period, continuous rain was observed for up to 12 days in the areas of both clusters. Dry weather (days without rain) lasted for 57 days and 19 days in the areas of cluster 1 and cluster 2, respectively. Although the mosquito abundance did not show a statistically significant difference based on meteorological phenomena in either cluster (Table S1), the negative effects of rainfall on the mosquito abundance were observed in cluster 2 (Fig. 5). The change rate of the mosquito abundance was significantly different before and after rainy weather (pairwise Mann–Whitney *U* test, *p* < 0.05) when there had been no rain before the period of rainy weather for more than 4 days.

### Effect of environmental variables on the mosquito occurrence prediction

**Fig. 6:**
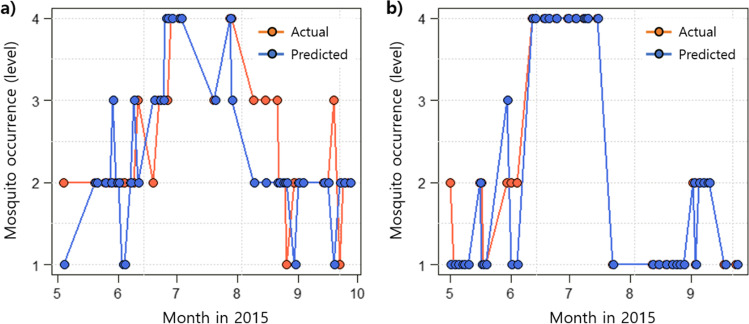
Examples of model performance with test datasets in 2015. **a** Site P in cluster 1, **b** site K in cluster 2

Based on the cumulative meteorological and landscape variables in the test datasets, the RF models predicted the mosquito occurrence levels in each cluster and showed a high prediction performance (Fig. 6). For the test dataset, the ACC was 0.895 and 0.870 for clusters 1 and 2, respectively, and the AUROC values were 0.827 and 0.808, respectively.

**Fig. 7:**
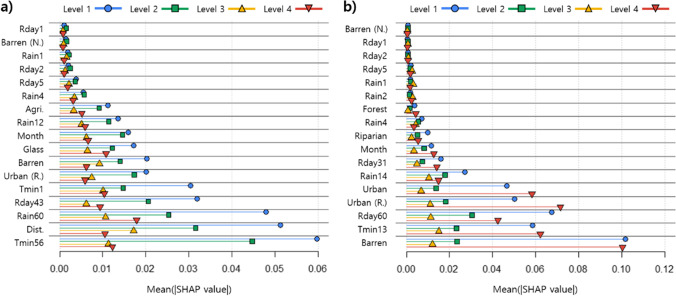
Variable importance in the RF models for cluster 1 (**a**) and cluster 2 (**b**). Importance was calculated as the mean absolute of the SHAP values. Abbreviations for the variables are given in Table 3

The influence of the environmental variables on mosquito occurrence was evaluated using SHAP values (Fig. 7). Variables such as the cumulative minimum temperature for 56 days, the cumulative rainfall for 60 days, and the cumulative number of rainy days for 43 days strongly affected the mosquito occurrence levels, especially the low level of mosquito occurrence in cluster 1. The distance to rivers, residential areas, and barren areas were also important landscape variables. In the RF model for cluster 2, the barren area was the most important variable, followed by the cumulative minimum temperature for 13 days, the cumulative number of rainy days for 60 days, and the residential area. These variables strongly affected the high level of mosquito occurrence in cluster 2.

**Fig. 8:**
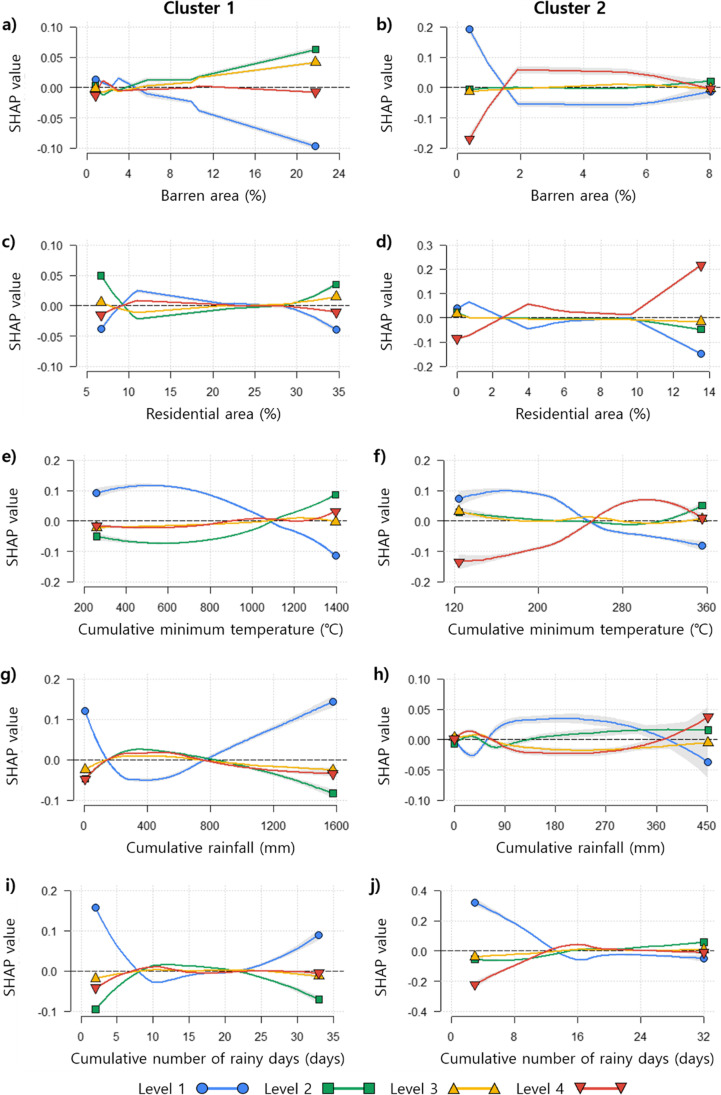
Partial dependence plots of RF models for each cluster (cluster 1, **a**, **c**, **e**, **g**, **i**; cluster 2, **b**, **d**, **f**, **h**, **j**). Partial dependence plots were drawn using the SHAP value. Among whole independent variables, only selected variables of high importance were presented. **a**–**b** Proportion of barren area, **c**–**d** proportion of residential area, **e**–**f** the cumulative minimum temperature for 56 (**e**) and 13 days (**f**), **g**–**h** the cumulative number of rainfall for 60 (**g**) and 14 days (**h**), **i**–**j** the cumulative number of rainy days for 43 (**i**) and 60 days (**j**). The lines display the local polynomial regression curve for the SHAP values to the dependent variables, and the gray area indicates the 95% confidence interval

The effect of environmental variables on the mosquito occurrence levels in both RF models was evaluated using partial dependence plots (PDPs) with SHAP values (Fig. 8). The responses of the SHAP values were different for the low and high mosquito levels in each model. With the increase in the proportion of barren area in both models, the SHAP values decreased for mosquito level 1 and increased for levels 2 and 3 in cluster 1, as well as for level 4 in cluster 2 (Fig. 8a–b). Meanwhile, the SHAP values increased for level 4, while decreasing for level 1 with an increase in the proportion of residential area (Fig. 8c–d). Under higher cumulative minimum temperatures, the mosquito occurrence shifted from low to high levels. In particular, the SHAP values for mosquito level 4 increased considerably in cluster 2 (Fig. 8e–f). The responses of the SHAP values to cumulative rainfall were higher in cluster 1 than in cluster 2. In the model for cluster 1, the SHAP values for level 1 were high, with low and high amounts of cumulative rainfall (Fig. 8g–h). This trend was also observed in the PDP for the cumulative number of rainy days. However, the effect of the cumulative number of rainy days was greater in the model for cluster 2 than for cluster 1 (Fig. 8i–j).

## Discussion

### Landscape effects

Mosquito occurrence is largely determined by the environmental conditions (Chaves et al. 2011; Cleckner et al. 2011; Kwon et al. 2015). In this study, it depended on the differences between these conditions in the landscapes being monitored. Twenty-one mosquito monitoring sites were classified into two clusters based on the landscape conditions. Cluster 1 was characterized by a non-waterside area with a residential area, whereas cluster 2 was characterized by the waterside with wetland and grass areas (Fig. 2). A higher mosquito abundance was detected in the waterside area than in the non-waterside area (Fig. 3). These results support previous findings showing that mosquitoes prefer wet habitats, with mosquito abundance having a positive association with the rates of grass area and wetland in their habitat (Chuang et al. 2011; Webb et al. 2016). In the present study, two different prediction models for the occurrence of mosquitoes were developed based on environmental variables.

### Meteorological conditions

Meteorological factors greatly affected the mosquito occurrence. The temperature has a major influence on species development and mortality rates (Beck-Johnson et al. 2017; Dosland et al. 2006), and rainfall provides a suitable habitat for mosquitoes (Chuang et al. 2011; Webb et al. 2016; Wilke et al. 2017). The minimum temperature is of particular importance for the development of poikilotherms, including mosquitoes (Higley and Haskell 2001; Lindblade et al. 2000). Therefore, the daily minimum temperature was employed in our prediction model by considering its ecological influence and covariance with other temperature variables.

The development of mosquitoes reflects the cumulative conditions in their environment, and their occurrence displays time-delayed responses to changes in the meteorological conditions (Hunter and Price 1998, Lebl et al. 2013; Park et al. 2003). In this study, the cumulative meteorological factors had a stronger correlation with mosquito abundance than the time-lagged variables (Fig. 4), highlighting the importance of their contribution to mosquito occurrence. Herein, the cumulative meteorological values were used to explain the delayed and cumulative effects on the mosquito occurrence in the prediction models.

Although most cumulative values of the meteorological variables were positively correlated with the mosquito abundance in both clusters, the strength and trend of the correlations were different between areas (i.e., clusters) (Fig. 4). The non-waterside area (cluster 1) showed a high correlation coefficient on high cumulative temperature days, whereas the waterside area (cluster 2) had a high coefficient on low cumulative days for the three temperature types. The effect of rainfall was similar to that of temperature.

Rainfall fluctuation negatively affects the growth rate of mosquito populations (Yang et al. 2008), and the number of rainy days is one of the factors influencing the mosquito occurrence (Valdez et al. 2017; Wilke et al. 2017). In this study, we observed a negative effect for rainfall on the mosquito abundance in waterside areas (Fig. 5). Rainfall patterns are an essential determinant of mosquito occurrence because they require standing water to survive and reproduce. However, rainfall beyond certain levels has a negative impact on mosquitoes by flushing them out (Benedum et al. 2018). In the present study, the rate of change in the mosquito abundance was significantly different under rainy conditions.

### Prediction model for mosquito occurrence

Both RF models for the waterside and the non-waterside areas in the metropolitan city showed good performance in the prediction of the mosquito occurrence. However, the contribution of environmental variables differed between the models based on the SHAP values (Fig. 7). The cumulative meteorological variables and the distance to the rivers were important factors in predicting the mosquito occurrence in the non-waterside area, whereas land cover variables and the cumulative meteorological variables were important in the waterside area. The SHAP values for each variable were high for low mosquito levels (levels 1 and 2) in the non-waterside area model while they were high for the high mosquito occurrence levels (levels 1 and 4) in the waterside area model. These results reflect the different environmental conditions of the study area in the models (Figs. 1–3). The SHAP approach revealed that low levels of mosquito occurrence in the non-waterside area were strongly affected by the meteorological and geographical conditions, whereas the low and high levels of mosquito occurrence in the waterside area were influenced by the land cover and the meteorological environments.

The relationship between the dependent variables and the mosquito occurrence levels could be explained using partial dependence plots with SHAP values (Fig. 8). The SHAP values for mosquito occurrence level 1 commonly decreased as a function of the proportion of barren area and the cumulative minimum temperature, presenting a high contribution of these factors in the prediction of high mosquito occurrence in the waterside and non-waterside areas. A low level of mosquito occurrence was predicted based on the effects of drought in both areas. When the cumulative rainfall and the number of rainy days were low, the SHAP values for mosquito level 1 were high. Heavy rain strongly affected the prediction of the lowest mosquito level in the model for the non-waterside area.

### Machine learning model and interpretation

Our results demonstrate that the RF model can be effectively used for the prediction of mosquito occurrence based on the landscape and meteorological factors. Machine learning methods such as RF do not require expert knowledge of the system involved because they are based on common machine learning algorithms, namely, ensemble learning (Wieland et al. 2021), which has exhibited superior performance in various research fields (Cuéllar et al. 2020; Kwon et al. 2015; Lee et al. 2021b).

In general, machine learning methods tend to lack interpretability. Various methods have been developed to overcome this limitation. Among them, SHAP has received considerable attention owing to its advantages, which are as follows (Abdollahi and Pradhan 2021, Lundberg and Lee 2017, Molnar 2022): (1) the SHAP approach is based on the Shapley values from game theory and satisfies three feature attributions, namely, local accuracy, missingness, and consistency; (2) the SHAP value can be calculated by a model-agnostic or model-specific estimation method ensuring a broad scope of application or fast calculation; and (3) although the SHAP method was developed for a local explanation of the model, a global explanation is also effectively possible. Therefore, the SHAP approach can be used in various ways according to its purpose, such as individual conditional expectation, feature importance, and feature dependence.

A considerable number of studies from various research fields have recently employed the SHAP method. Wieland et al. (2021) used it to evaluate the importance of independent variables in mosquito habitat models. Cha et al. (2021) reported that the SHAP method could be used to evaluate models and provide management support by determining the contribution of environmental variables in the species distribution model. Through the SHAP method, our RF models accurately described the relationship between mosquito occurrence and the environment in both waterside and non-waterside areas of the metropolitan city, with plausible interpretation.

## Conclusions

Many local governments in South Korea have introduced mosquito forecasting systems without sufficient consideration of the habitat conditions of mosquitos. However, mosquito occurrence is influenced by various environmental factors, such as landscape and meteorological variables. In this study, we analyzed mosquito occurrence patterns in relation to the meteorological factors within the specific landscape environment and developed a model for the prediction of mosquito occurrence using RF. Mosquito occurrence patterns and their relationship with meteorological factors differed between the waterside and the non-waterside areas. The mosquito population in the waterside area showed a negative association with rainfall in the case of excessive rainfall. The RF models for both areas exhibited good performance in predicting the mosquito occurrence levels, both in terms of accuracy and AUROC. The relationship between the mosquito occurrence levels and the environmental factors was explained through variable importance and partial dependence plots using SHAP values. The waterside area was influenced to a greater extent by the meteorological and land cover variables than the non-waterside area. Therefore, mosquito control strategies should consider the effects of landscape and meteorological conditions, including the temperature, rainfall, and the landscape heterogeneity. Our study revealed that interpretable machine learning methods can contribute to the evaluation of environmental factors on mosquito occurrence as well as the development of a mosquito forecasting system and public health.

## Supplementary Information

Below is the link to the electronic supplementary material.

Supplementary file1 (PDF 169 KB)

## Data Availability

Available under request.
