# Supplementary material for: Interpretable machine learning approach to analyze the effects of landscape and meteorological factors on mosquito occurrences in Seoul, South Korea
Source: Environ Sci Pollut Res Int. 2022 Jul 28;30(1):532–46. doi: 10.1007/s11356-022-22099-5 (PMC9813121; doi:10.1007/s11356-022-22099-5)
Supplement: Supplementary file 1 — Supplementary file1 (PDF 169 KB) [file 11356_2022_22099_MOESM1_ESM.pdf]

Electronic supplementary information

Interpretable machine learning approach to analyze the effects of landscape and meteorological factors on mosquito occurrences in Seoul, South Korea

Dae-Seong Lee, Da-Yeong Lee, Young-Seuk Park

Department of Biology, Kyung Hee University, Seoul 02447, Republic of Korea

During the study period, mosquito abundance was not significantly different before a rainy weather (amount of rainfall = 0 mm) and rainy days (i.e., the first day of rain; the amount of rainfall > 0 mm), between before and after the rainy weather (amount of rainfall = 0 mm), as well as between the first (after the rainy weather) and last day (before the rainy weather) of a dry weather (pairwise Mann-Whitney U test, $p > 0.1$ in both clusters) (Table S1).

Table S1. Effects of meteorological phenomena on mosquito abundance in each cluster.

Meteorological phenomena			Median value of abundance	
			**Cluster 1**	**Cluster 2**
Before a rainy weather (A)	&	First day of the rainy weather (B)	(A) 24.2, (B) 20.7 (N= 89, p = 0.531)*	(A) 129.6, (B) 141.3 (N= 74, p = 0.435)
Before a rainy weather (A)	&	After the rainy weather (B)	(A) 24.2, (B) 22.3 (N= 89, p = 0.173)	(A) 131.5, (B) 117.7 (N= 75, p = 0.484)
First day of a dry weather (A)	&	Last day of the dry weather (B)	(A) 22.1, (B) 21.2 (N= 63, p = 0.585)	(A) 114.0, (B) 125.8 (N= 47, p = 0.331)

* N: number of compared samples; *p*: *p*-value of the pairwise Mann-Whitney U test.
